# Insulin resistance and brain health in midlife: a potential dementia prevention target

**DOI:** 10.1016/j.ebiom.2026.106448

**Published:** 2026-08-21

**Authors:** Rohith Erukulla, Graham Reid, Courtney Yotter, Maria-Elini Dounavi, Audrey Low, John T. O'Brien, Craig Ritchie, Brian Lawlor, Lorina Naci, Paresh Malhotra, Ivan Koychev

**Affiliations:** a University of Oxford Department of Psychiatry, Warneford Hospital, Warneford Ln, Headington, Oxford, OX3 7JX, United Kingdom; b Department of Psychiatry, University of Cambridge School of Clinical Medicine, Cambridge, CB2 0SP, United Kingdom; c Edinburgh Dementia Prevention, Centre for Clinical Brain Sciences, University of Edinburgh, Edinburgh, EH4 2XU, United Kingdom; d Global Brain Health Institute, Trinity College Dublin, Lloyd Building, Dublin, D02 X9W9, Ireland; e Trinity College Institute of Neuroscience, Trinity College Dublin, Lloyd Building, Dublin, D02 X9W9, Ireland; f Imperial College London, UK Dementia Research Institute Care Research and Technology Centre, London, W12 0BZ, United Kingdom

**Keywords:** Diabetes, Dementia, Insulin resistance, White matter hyperintensity, Cerebral blood flow

## Abstract

**Background:**

Insulin resistance is recognised as a midlife risk factor for cognitive decline, yet the pathways linking metabolic dysfunction to early brain changes remain unclear.

**Methods:**

We cross-sectionally analysed 355 cognitively normal adults from the PREVENT cohort to test associations between insulin sensitivity (Homoeostatic Model Assessment for Insulin Resistance; HOMA-IR), frontal white matter hyperintensities (WMH), cerebral blood flow (CBF), hippocampal volume, and cognition.

**Findings:**

Multivariable regression showed higher log-transformed HOMA-IR was associated with greater frontal WMH burden (β = 0.118, p = 0.026) and lower global cognitive performance (β = −0.132, p = 0.012). Decreased insulin sensitivity was not associated with global CBF (β = −0.019, p = 0.74). Adjusting for WMH burden, hippocampal volume, and CBF, lower insulin sensitivity remained associated with lower global cognition (β = −0.123, p = 0.021). Structural equation modelling (n = 328) demonstrated a direct negative association between higher HOMA-IR and cognition (β = −0.055, p = 0.042) and trended toward higher vascular burden (p = 0.06). The indirect pathway through vascular burden was non-significant (p = 0.23), as vascular burden, hippocampal volume, and CBF did not associate with cognition.

**Interpretation:**

Decreased insulin sensitivity was linked to early small vessel disease and measurable cognitive differences, suggesting the cognitive association was largely direct rather than explained by vascular injury, hippocampal atrophy, or global perfusion. These findings point toward partially parallel metabolic and vascular pathways rather than a sequential process. These cross-sectional associations suggest that insulin sensitivity warrants investigation as a modifiable midlife factor for cognitive health; interventional studies are needed to determine whether targeting it preserves cognition before neurodegenerative markers emerge.

**Funding:**

MRC Dementias Platform UK, NIHR, Alzheimer's Society, Alzheimer's Association, Race Against Dementia, and HRB.

**Research in context**

**Evidence before this study**

We searched PubMed and MEDLINE for articles published in English between January 1, 2010, and May 1, 2026, using the search terms (“insulin resistance” OR “metabolic syndrome”) AND (“white matter hyperintensities” OR “cerebral blood flow” OR “cognition”). Prior work connects insulin signalling deficits to amyloid and tau biology, while epidemiological studies show that metabolic dysfunction predicts poorer executive function and processing speed. White matter hyperintensities are a known consequence of vascular injury and correlate with metabolic risk. Cerebral blood flow reductions have also been proposed as a mediator between metabolic dysfunction and brain ageing but findings in midlife cohorts remain inconsistent. Few studies have examined insulin resistance, vascular injury, perfusion, hippocampal structure, and cognition together in a single framework.

**Added value of this study**

Using a unified multivariable regression and structural equation modelling framework in a cognitively normal midlife cohort, this study demonstrates that decreased insulin sensitivity is associated with greater frontal white matter hyperintensity burden, larger hippocampal volume, and lower global cognitive performance. Notably, the association between insulin sensitivity and cognition remained significant after adjustment for WMH burden, hippocampal volume, and cerebral blood flow, suggesting that metabolic dysfunction relates to cognitive variability beyond measurable vascular injury, brain structure, or global perfusion. Structural equation modelling further supported a direct pathway linking decreased insulin sensitivity to cognition, with only a trend–level association with latent vascular burden and no significant indirect vascular pathway.

**Implications of all the available evidence**

Metabolic dysfunction may be associated with brain health earlier than previously assumed, and insulin resistance was associated with both vascular and cognitive measures before neurodegeneration became evident on MRI. Strategies targeting insulin sensitivity may warrant investigation as a route to modifying dementia risk during midlife, pending interventional evidence. The absence of mediation through hippocampal atrophy or cerebral perfusion, together with the persistence of a direct association between decreased insulin sensitivity and cognition, suggests that decreased insulin sensitivity influences cognition through mechanisms that are at least partly independent of classical neurodegenerative markers, emphasising the need to treat metabolic health as a core component of dementia prevention.

## Introduction

Dementia develops over decades, and midlife has emerged as a critical window for identifying modifiable risk factors that influence later cognitive decline. Among these, insulin resistance (IR) is increasingly recognised as an upstream contributor to brain ageing. Type 2 diabetes is consistently associated with accelerated cognitive decline and elevated dementia risk, suggesting that metabolic dysfunction may affect neural integrity well before clinical disease onset.^1^^,^^2^

Insulin signalling plays an essential role in central nervous system function, influencing synaptic plasticity, neuronal survival, and cerebral glucose metabolism.^3^^,^^4^ Impaired insulin signalling has been linked to tau phosphorylation, amyloid pathology, and reduced cerebral metabolism, supporting a mechanistic connection between metabolic dysfunction and neurodegeneration. Intranasal insulin improves memory and attention in early AD.^5^^,^^6^ Recognising these broad neural effects, the field increasingly conceptualises this phenomenon as dysregulated brain insulin signalling rather than solely an issue of glucose mishandling.^7^ Decreased insulin sensitivity has also been tied to deficits in memory, executive function, and processing speed, even in populations without diabetes, indicating that metabolic disruption itself may influence cognition.^8^

Vascular mechanisms represent one proposed pathway linking IR to cognitive decline. White matter hyperintensities (WMHs), markers of small vessel disease, are associated with executive dysfunction and increased dementia risk.^9^ IR has been linked to greater WMH burden, suggesting that metabolic dysfunction may contribute to cerebrovascular injury.^10^ Altered cerebral blood flow (CBF) has also been proposed as a potential pathway linking metabolic dysfunction to cognitive outcomes, although findings, particularly in midlife cohorts, have been inconsistent and may reflect complex or indirect relationships.^11^^,^^12^

Despite these converging lines of evidence, few studies have examined insulin resistance, white matter injury, cerebral perfusion, hippocampal structure, and cognition simultaneously within a unified multivariate framework in cognitively normal midlife adults. Midlife represents a critical window during which metabolic and vascular processes present cross-sectional associations with brain health long before dementia manifests.^13^^,^^14^ Importantly, cognitive differences related to decreased insulin sensitivity in midlife may represent reversible metabolic states rather than permanent neurodegenerative trajectories. Determining whether insulin resistance affects cognition directly, or indirectly through vascular injury, perfusion changes, or neurodegenerative processes, has important implications for early intervention strategies.

The present study leverages the PREVENT Dementia cohort of middle-aged adults (40–59 years) to examine associations between insulin resistance and (1) frontal white matter hyperintensity burden, (2) hippocampal volume, (3) cerebral blood flow, and (4) cognitive performance. Using multivariable regression and confirmatory structural equation modelling (SEM), we test whether insulin sensitivity relates to cognition directly or indirectly through vascular burden, perfusion, or neurodegenerative pathways. We hypothesised that decreased insulin sensitivity would be associated with greater vascular burden and altered perfusion, and that insulin sensitivity may relate to cognitive performance both directly and indirectly within a multivariate framework.

## Methods

### Participants

The PREVENT Research Program established a cohort to study early brain changes in middle-aged individuals.^15^ Participants aged 40–59 years, with or without a parent with dementia, were recruited from the West London Mental Health NHS Trust database and the Join Dementia website across London, Edinburgh, Cambridge, and Oxford. The Dublin cohort was excluded from this study since the PREVENT MRI ASL protocol was not followed. While the PREVENT Dementia programme is designed to collect longitudinal testing for various biological and cognitive variables, the present study relies exclusively on cross-sectional data captured at the baseline visit to evaluate early midlife associations. At recruitment, participants self-reported cognitive health and showed no dementia signs. The Addenbrooke's Cognitive Assessment III (ACE-III) was administered to a subset during baseline visits as it was introduced midway through data collection. Further details on the PREVENT cohort protocol are available.^15^^,^^16^

Participants in the PREVENT cohort with complete MRI and arterial spin labelling (ASL) data relevant to this study, excluding incidental MRI findings, were identified. Sample size was determined by the availability of complete baseline imaging and metabolic data within the cohort, rather than an a priori power calculation for this secondary analysis. The participants' demographic data were recorded, including age, sex assigned at birth (self-reported), BMI, years of education, and APOE4 carriership. While race and ethnicity data were collected within the broader PREVENT Dementia programme, these demographic variables were not included in the specific data extract provided for this analysis and thus were not analysed.

### Insulin sensitivity

At the first appointment of the PREVENT cohort study, the fasting glucose and fasting insulin concentrations of the individuals were determined in plasma blood samples. This information was then used to calculate IR using the Homoeostatic Model Assessment for Insulin Resistance (HOMA-IR) according to the following formula: fasting plasma glucose (mmol/l) times fasting serum insulin (mU/l) divided by 22.5.^17^ Given the relatively healthy status of this cohort (mean BMI = 26.79), clinical insulin resistance was rare. Therefore, HOMA-IR was utilised as a continuous measure to capture the full spectrum of insulin sensitivity rather than a binary clinical diagnosis.

### Cognitive assessments

Participants were assessed for neuropsychological measures, including attention, memory, language, and visuospatial abilities, using the COGNITO battery, a computerised assessment that records responses via a touchscreen.^18^ This study examined Stroop test scores, reaction times, processing speed, executive function, delayed recall, and verbal fluency (semantic and phonemic). Processing speed was calculated by deducing reaction time from response latency, while executive function was a latent construct derived from semantic and phonemic verbal fluency.^19^ Visuospatial orientation was evaluated using the 4 Mountains Test, requiring participants to recall the spatial configuration of a computer-generated landscape from a shifted viewpoint.^20^

### Imaging acquisition

Participants were scanned using 3T Siemens scanners (specific models: Verio, PRISMA, Prisma Fit, Skyra). A 3D T1 weighted magnetisation prepared rapid gradient-echo (MPRAGE) image (TR = 2.3 s, TE = 2.98 ms, 160 slices, flip angle = 9°, voxel size = 1 mm^3^ isotropic), a fluid-attenuated inversion recovery (FLAIR) scan (TR = 9 s; TE = 94 ms, 27 slices, voxel size = 0.4 × 0.4 × 4 mm, TI = 2500 ms; flip angle = 150°), and an ASL dataset (PICORE Q2TIPS, 50 pairs of control/tag images, one calibration image, TR = 2.5 s/TE = 11 ms, inversion time = 1.8 s, bolus duration = 700 or 1675 ms, voxel size 3.0 × 3.0 × 6.0 mm, 14 slices, flip angle = 90°) were taken.^16^

### Image preprocessing

The T1-weighted images were processed using FreeSurfer version 7.1,^21^ while the ASL data were analysed using the oxford_asl pipeline within the FSL software package.^22^ The WMH processing of FLAIR images utilised SPM.^23^ To account for site-related variability across scanners, imaging-derived measures were harmonised using the ComBat method, with age, sex, years of education, APOE4 carriership, and family history of dementia included as biological covariates. Simplified versions of the preprocessing protocols are described below:

a. CBF

Preprocessing of the ASL data was conducted using the Bayesian Inference for ASL MRI toolbox in the FMRIB Software Library (FSL).^24^ ASL scans were motion-corrected with FSL's MCFLIRT, calibrated using proton density acquisition, and corrected for partial volume effects (PVC). CBF was quantified with the Buxton ASL kinetic model (Buxton et al., 1998; Chappell et al., 2011),^25^^,^^26^ accounting for differences in bolus duration. CBF maps were analysed in both native space and normalised MNI space, with derived values specified for grey matter (GM) at 20% and 50% GM content thresholds to accurately reflect GM-specific perfusion. Further details on the processing pipeline have been previously published.^21^

b. Hippocampal volume

Right hippocampal volumes were estimated by converting each participant's T1-weighted image to Talairach space and applying the pre-described procedure.^27^ Volumes were normalised to total intracranial volume (TIV) to account for individual differences in head size.

c. WMH quantification

The Statistical Parametric Mapping 12 suite was used to create WMH lesion maps from the FLAIR MRI data using an automated script. All lesion maps were reviewed by an experienced rater, blinded to clinical data, who manually delineated WMH locations to ensure quantification accuracy. Lesion maps were then normalised to MNI space to generate voxel-wise WMH probability maps.

Total and regional WMH volumes, including periventricular, deep, and lobar regions (frontal, parietal, temporal, and occipital), were extracted for each participant. For the primary analyses, frontal WMH burden was selected a priori as the primary vascular marker due to its established sensitivity to metabolic risk factors and cognitive performance in midlife.^28^^,^^29^ This region was prioritised due to its greater variability in midlife, established sensitivity to metabolic and small vessel risk factors, and well-documented associations with executive and global cognitive performance. Frontal WMH burden was calculated as the sum of left and right frontal WMH volumes normalised by TIV to account for differences in head size. Further details have been previously published.^23^

To evaluate whether observed associations reflected global rather than regionally specific small vessel disease, total WMH burden normalised to TIV was examined as a pre-specified sensitivity analysis using the same covariates and analytic framework.

### Ethics

Written informed consent was obtained from all participants. The PREVENT Dementia programme received multi-site ethical approval from the UK London-Camberwell St Giles National Health Service (NHS) Research Ethics Committee (REC reference: 12/LO/1023, IRAS project ID: 88938), which operates according to the 2024 Declaration of Helsinki. All substantial protocol amendments have been reviewed by the same ethics committees and favourable opinion was granted before implementation at sites.

### Statistics

All statistical analyses were conducted in Python using the statsmodels and semopy packages. Continuous variables were converted to numeric format where necessary. Two-sided p-values <0.05 were considered statistically significant. Because analyses were restricted to a priori, hypothesis-driven models targeting predefined outcomes, correction for multiple comparisons was not applied.

Insulin sensitivity was indexed using HOMA-IR and transformed using a log (1 + x) transformation to reduce skewness and improve the normality of residuals for regression and SEM. A global cognition composite was created for regression analyses by z-scoring delayed recall, executive function, Stroop performance, semantic verbal fluency, and phonemic verbal fluency, and computing the mean of these standardised scores.

Primary analyses were conducted using ordinary least squares (OLS) regression with heteroscedasticity-robust standard errors (HC3). Predictor variables were standardised using z-score scaling to facilitate interpretation and comparability of effect sizes. Age, sex, and APOE4 status were included as covariates in all models. BMI and WHR were not included in primary analyses due to collinearity with HOMA-IR.

Five regression models were specified: (1) frontal WMH ratio regressed on log-transformed HOMA-IR and CBF; (2) hippocampal ratio regressed on log-transformed HOMA-IR; (3) CBF regressed on log-transformed HOMA-IR; (4) global cognition regressed on log-transformed HOMA-IR and education; and (5) global cognition regressed on frontal WMH ratio, hippocampal ratio, cerebral blood flow, log-transformed HOMA-IR, and education.

SEM was subsequently performed in participants with complete data to evaluate relationships among insulin sensitivity, vascular burden, hippocampal volume, cerebral blood flow, and cognition within a single multivariate framework. All SEM variables were standardised prior to estimation. A latent Vascular construct was defined by left and right frontal WMH ratios, and a latent Cognition construct was defined by delayed recall, executive function, Stroop performance, semantic verbal fluency, and phonemic verbal fluency. Structural paths were specified from log-transformed HOMA-IR to vascular burden, hippocampal ratio, and CBF. Direct paths were specified from vascular burden, hippocampal ratio, CBF, and log-transformed HOMA-IR to cognition. Age was included as a predictor of vascular burden, hippocampal ratio, CBF, and cognition, and education was included as a predictor of cognition. Model fit was evaluated using the Comparative Fit Index (CFI), Tucker–Lewis Index (TLI), Root Mean Square Error of Approximation (RMSEA), and Goodness-of-Fit Index (GFI).

### Role of funders

IK declares funding for this work through the Medical Research Council Dementias Platform UK grant (MR/T033371/1) and the National Institute for Health and Care Research (personal award NIHR301616 and Oxford Health Biomedical Research Centre). The PREVENT dementia Programme is funded by the Alzheimer's Society (grant numbers 178, 264 and 329), Alzheimer's Association (grant number TriBEKa-17-519007), and philanthropic donations. JOB is supported by the NIHR Cambridge Biomedical Research Centre, MD by an Alzheimer's Society Fellowship, and AL by a Race Against Dementia Fellowship. LN was funded by a Health Research Board Individual Led Project Grant (ILP-POR-2024-057). The funders had no role in the study design, data collection, data analyses, interpretation, or writing of the report.

## Results

A total of 355 participants from the PREVENT cohort met study criteria. Sample sizes varied slightly across models due to missing data: 341 participants were included in the vascular regression, 331 in cognitive models, 342 in hippocampal analyses, and 328 in the structural equation modelling (SEM), which included CBF measures. Descriptive statistics for demographic and cognitive variables are presented in Table 1. In sensitivity analyses replacing frontal WMH with total WMH burden, insulin sensitivity was not significantly associated with total WMH.

**Table 1:** Demographic, clinical, and neuroimaging characteristics of the study sample.

Characteristic	N	Mean	SD
Age	355	51.45	5.45
Sex (Female) (%)	355	67	–
Education (Years)	355	16.6	3.4
BMI	354	26.79	5.04
Waist-to-Hip ratio	355	0.87	0.1
Fasting insulin	349	57.25	43.01
HOMA-IR (Raw)	342	1.06	0.8
HOMA-IR (Log-Transformed)	342	0.67	0.3
Frontal WMH ratio	354	7.80E-07	1.17E-06
Hippocampal ratio	355	2.82E-03	2.76E-04
Global CBF	355	47.95	7.08
Global cognition (Z)	344	1.06E-03	0.58
Delayed recall	344	6.89	1.53
Executive function	344	9.2	2.09
Stroop performance	343	22.44	2.23
Semantic fluency	343	16.87	3.96
Phonemic fluency	344	11.6	4.07

Summary of the cohort used in the structural equation model. Neuroimaging measures (Frontal WMH, Hippocampal Volume) are normalised by Total Intracranial Volume (TIV). Global Cognition represents a composite Z-score derived from delayed recall, executive function, Stroop, and fluency tasks.

### Primary regression analyses

#### Insulin sensitivity and frontal WMH burden

In multivariable linear regression (n = 341), higher log-transformed HOMA-IR was significantly associated with greater frontal white matter hyperintensities (WMH) ratio (β = 0.118, p = 0.026 [Multivariable linear regression]; Table 2), indicating that decreased insulin sensitivity was related to higher WMH burden.The overall model was statistically significant (F = 6.11, p = 0.0005 [Multivariable linear regression]; R^2^ = 0.052).

**Table 2:** Standardised multiple linear regression models evaluating associations between insulin sensitivity, brain health, and global cognition.

	Predictor	Std. beta	SE	p-value
**Model 1: Frontal WMH**	Log-HOMA (Decreased Insulin Sensitivity)	0.118	0.053	0.026
Overall Model Fit: F = 6.11, R^2^ = 0.052, p = 0.0005	Age	0.193	0.053	<0.0001
	Cerebral Blood Flow	−0.028	0.053	0.61
	Sex (Female)	−0.072	0.054	0.18
	APOE4 Status	0.015	0.054	0.79
**Model 2: Hippocampal Volume**	Log-HOMA	0.121	0.053	0.024
Overall Model Fit: F = 1.48, R^2^ = 0.009, p = 0.23	Age	0.006	0.054	0.91
	Sex (Female)	0.238	0.054	<0.0001
	APOE4 Status	−0.037	0.053	0.49
**Model 3: Global CBF**	Log-HOMA	−0.019	0.055	0.74
Overall Model Fit: F = 0.81, R^2^ = 0.005, p = 0.45	Age	0.077	0.055	0.17
	Sex (Female)	0.054	0.055	0.32
	APOE4 Status	0.069	0.055	0.21
**Model 4: Cognition (Direct)**	Log-HOMA	−0.132	0.053	0.012
Overall Model Fit: F = 12.50, R^2^ = 0.103, p < 0.0001	Age	−0.187	0.053	<0.0001
	Education	0.194	0.053	<0.0001
	Sex (Female)	0.194	0.052	<0.0001
	APOE4 Status	−0.017	0.052	0.75
**Model 5: Cognition (Fully Adjusted)**	Frontal WMH Ratio	−0.059	0.054	0.28
Overall Model Fit: F = 6.54, R^2^ = 0.108, p < 0.0001	Hippocampal Volume	−0.058	0.054	0.29
	Cerebral Blood Flow	−0.025	0.052	0.63
	Log-HOMA	−0.123	0.053	0.021
	Education	0.175	0.053	<0.0001
	Age	−0.167	0.054	0.002
	Sex (Female)	0.202	0.054	<0.0001
	APOE4 Status	−0.023	0.052	0.66

This table presents the standardised beta coefficients, standard errors (SE), and p-values derived from five distinct multiple linear regression models. Models 1 through 3 examine the influence of Log-HOMA (Decreased Insulin Sensitivity) and age on neuroimaging markers (Frontal WMH Ratio, Hippocampal Volume, and Global Cerebral Blood Flow). Models 4 and 5 assess predictors of Global Cognition, with Model 4 evaluating the direct effects of decreased insulin sensitivity, age, and education, and Model 5 representing a fully adjusted model that incorporates the brain health mediators. All continuous variables were standardised prior to analysis to yield comparable effect sizes. Statistical significance is indicated by p < 0.05.

#### Insulin sensitivity and hippocampal volume

Higher log-transformed HOMA-IR was significantly associated with larger hippocampal volume normalised to intracranial volume (n = 342; β = 0.121, p = 0.024 [Multivariable linear regression]). However, the overall model was not statistically significant (F = 1.48, p = 0.23 [Multivariable linear regression]; R^2^ = 0.009). Therefore, this individual predictor relationship cannot be reliably interpreted and should be viewed with caution.

#### Insulin sensitivity and global cerebral blood flow

Log-transformed HOMA-IR was not significantly associated with global cerebral blood flow (n = 342; β = −0.019, p = 0.74 [Multivariable linear regression]). The overall model was not statistically significant (F = 0.81, p = 0.45 [Multivariable linear regression]; R^2^ = 0.005).

#### Insulin sensitivity and global cognition

In the primary cognitive model (n = 331), higher log-transformed HOMA-IR was significantly associated with lower global cognitive performance (β = −0.132, p = 0.012 [Multivariable linear regression]). The model was statistically significant (F = 12.50, p < 0.0001 [Multivariable linear regression]; R^2^ = 0.103).

In the fully adjusted model additionally including frontal WMH ratio, hippocampal volume, and cerebral blood flow (n = 330), higher log-transformed HOMA-IR remained significantly associated with lower global cognitive performance (β = −0.123, p = 0.021 [Multivariable linear regression]). Frontal WMH ratio (β = −0.059, p = 0.28 [Multivariable linear regression]), hippocampal volume (β = −0.058, p = 0.29 [Multivariable linear regression]), and cerebral blood flow (β = −0.025, p = 0.63 [Multivariable linear regression]) were not significantly associated with cognition in the fully adjusted model. The full model was statistically significant (F = 6.54, p < 0.0001 [Multivariable linear regression]; R^2^ = 0.108).

### Structural equation modelling

**Fig. 1:**
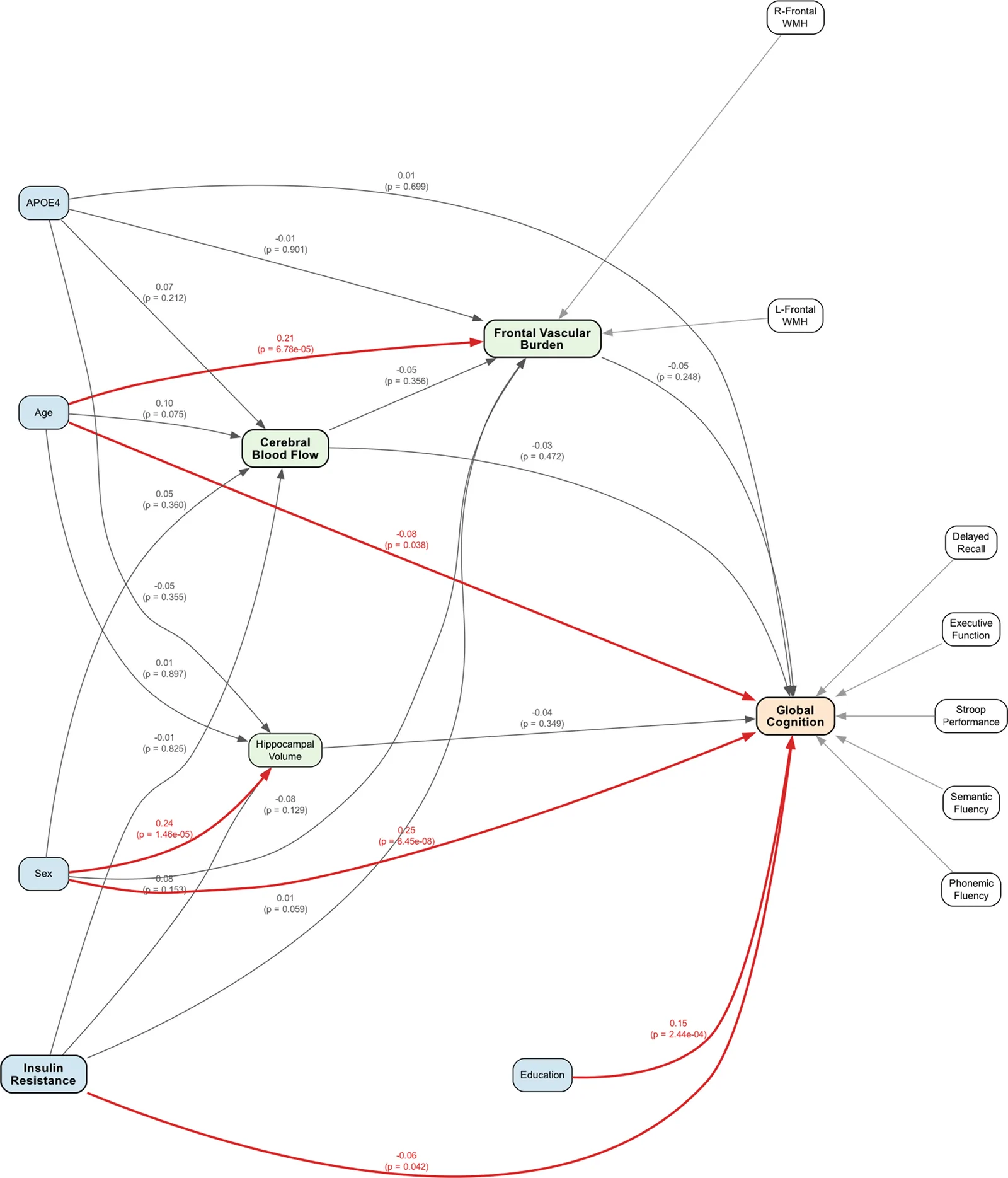
**Path analysis of the impact of decreased insulin sensitivity on brain structure and cognitive function.** Standardised path diagram illustrating the relationships between Log-HOMA (Decreased Insulin Sensitivity), markers of brain health, and Global Cognition. Red lines indicate statistically significant paths (p < 0.05), with standardised coefficients and p-values shown. Grey lines represent the latent variable definitions and black lines represent non-significant structural paths.

The SEM included 328 participants with complete data (See Fig. 1). Model fit indices indicated good fit (CFI = 0.960; TLI = 0.944; RMSEA = 0.048; GFI = 0.913 [Structural equation modelling]).

Within the structural model, higher log-transformed HOMA-IR was directly associated with poorer global cognition (β = −0.055, p = 0.042 [Structural equation modelling]). HOMA-IR showed a trend–level association with frontal vascular burden (β = 0.097, p = 0.06 [Structural equation modelling]) but was not associated with hippocampal volume (β = 0.079, p = 0.15 [Structural equation modelling]) or cerebral blood flow (β = −0.023, p = 0.67 [Structural equation modelling]). Age was significantly associated with higher vascular burden (β = 0.222, p < 0.0001 [Structural equation modelling]) and lower cognition (β = −0.086, p = 0.005 [Structural equation modelling]) but was not significantly associated with hippocampal volume (β = −0.005, p = 0.93 [Structural equation modelling]) or CBF (β = 0.088, p = 0.11 [Structural equation modelling]). Education was positively associated with cognition (β = 0.097, p = 0.002 [Structural equation modelling]).

Frontal vascular burden (β = −0.033, p = 0.23 [Structural equation modelling]), hippocampal volume (β = −0.012, p = 0.62 [Structural equation modelling]), and CBF (β = −0.021, p = 0.40 [Structural equation modelling]) were not significantly associated with cognition within the model. Consequently, the indirect pathway from insulin sensitivity to global cognition through frontal vascular burden was not statistically significant (indirect effect β = −0.003, 95% CI: −0.009 to 0.003 [Structural equation modelling]).

## Discussion

This study applied a unified multivariate and structural equation modelling framework to examine the pathways linking insulin sensitivity to cerebrovascular injury, brain structure, perfusion, and cognition in a cognitively normal midlife cohort. Across analytic approaches, two consistent findings emerged. First, decreased insulin sensitivity (indicated by higher HOMA-IR) was associated with greater frontal white matter hyperintensities (WMH) burden in multivariable models. Second, decreased insulin sensitivity demonstrated a direct negative association with global cognition in both multivariable regression and structural equation modelling. Notably, this association remained significant in a fully adjusted regression model including WMH burden, hippocampal volume, and CBF, suggesting that the cognitive associations of decreased insulin sensitivity are not fully explained by vascular injury, brain atrophy, or global perfusion differences. In contrast, hippocampal volume and global CBF were not significantly associated with cognition. Together, these findings support a model in which metabolic dysfunction in midlife is linked to early cerebrovascular injury and measurable cognitive differences through partially parallel, rather than strictly sequential, pathways.

The association between decreased insulin sensitivity and frontal WMH burden supports prior literature linking metabolic dysfunction to small vessel disease and white matter injury.^30^^,^^31^ In sensitivity analyses, this relationship was not observed when total WMH burden was examined, suggesting that decreased insulin sensitivity in midlife may relate to regionally selective rather than diffuse small vessel changes. Given the relatively low overall WMH burden in this cognitively normal cohort, frontal predominance may reflect early vascular vulnerability before more generalised white matter involvement becomes apparent. Frontal WMHs are well-established markers of small vessel pathology and are strongly associated with executive dysfunction and processing inefficiency in ageing populations.^29^ The localisation to frontal regions is notable, as these long association fibres may be particularly vulnerable to metabolic and vascular stress during midlife.^32^

However, while decreased insulin sensitivity showed a trend–level association with the latent vascular burden factor in SEM, vascular burden itself did not significantly predict cognition. This indicates that vascular burden did not explain the association between decreased insulin sensitivity and cognition in this cross-sectional sample. Consistent with this pattern, prior longitudinal work has shown that midlife insulin resistance predicts later cognitive decline even in the absence of substantial MRI-defined vascular lesion burden.^33^ Together, these findings indicate that small-vessel injury may represent an early parallel consequence of metabolic dysfunction rather than the primary driver of cognitive variability at this stage.

The most robust and clinically meaningful finding was the direct association between decreased insulin sensitivity and global cognition. In multivariable regression adjusting for age and education, decreased insulin sensitivity was associated with lower global cognitive performance, and this relationship persisted after additionally adjusting for frontal WMH burden, hippocampal volume, and CBF. The persistence of this effect in both regression and SEM frameworks indicates that insulin sensitivity-related cognitive differences are not fully attributable to measurable cerebrovascular injury, structural atrophy, or global perfusion. The latent cognition construct captured shared variance across multiple cognitive domains, suggesting that metabolic dysfunction relates to broad neural efficiency rather than isolated domain-specific impairment. These findings fit with hypotheses that disrupted insulin signalling can interfere with synaptic plasticity, neuronal energy use, and the stability of large-scale brain networks long before any clear neurodegenerative changes emerge.^7^^,^^8^^,^^34^ The cohort's high educational attainment could have conferred cognitive reserve that delays the clinical expression of early neural changes associated with decreased insulin sensitivity. Higher education is widely used as a proxy for cognitive reserve and is associated with better preserved cognition despite accumulating neuropathology, which may have reduced the sensitivity of global cognitive measures in this midlife sample.^35^

In contrast, the overall multivariable model predicting hippocampal volume from insulin sensitivity was not statistically significant, and hippocampal volume did not predict cognition in the SEM. This pattern suggests that structural neurodegeneration may not yet be detectable in midlife individuals with elevated metabolic risk. It is plausible that metabolic dysfunction initially affects neural function and network efficiency before measurable volumetric changes emerge, particularly in cognitively unimpaired cohorts with relatively preserved brain structure.^36^^,^^37^ Longitudinal follow-up will be necessary to determine whether persistently decreased insulin sensitivity accelerates later hippocampal atrophy or memory decline.

Global CBF was not associated with insulin sensitivity, vascular burden, hippocampal volume, or cognition in either regression or SEM analyses. In a relatively healthy midlife cohort, autoregulatory mechanisms may preserve overall cerebral perfusion despite rising metabolic risk. Whole-brain CBF appears to be an insensitive summary metric in this context. Global measures can fail to reflect regional perfusion differences and metabolic–blood flow decoupling that have been documented in metabolic and ageing samples, even when regional hypoperfusion and IR-related effects are present in frontal, temporal, and other cortex regions. Regional CBF alterations have been linked with metabolic risk and cognitive outcomes despite minimal changes in global CBF, suggesting that spatially resolved perfusion measures may be more sensitive to early metabolic–vascular dysfunction.^38^

The SEM demonstrated strong model fit, supporting the validity of the latent structure linking metabolic, vascular, and cognitive domains. The direct insulin sensitivity-to-cognition pathway remained significant after simultaneously modelling vascular burden, hippocampal structure, and perfusion, reinforcing a framework in which decreased insulin sensitivity is associated with both vascular and neural measures that are at least partially independent in midlife. This parallel-pathway interpretation may explain why WMH burden is detectable yet does not fully account for cognitive differences at this preclinical stage.

Several limitations warrant consideration. The cross-sectional design precludes causal inference and does not establish the temporal ordering necessary to support a definitive mechanistic interpretation of mediation pathways. Consequently, these structural relationships describe current, cross-sectional associations, and our findings should be viewed as preliminary and hypothesis-generating. Effect sizes were modest, which is expected in a cognitively unimpaired midlife sample with relatively low vascular and neurodegenerative burden. Perfusion analyses relied on global CBF rather than regional measures, and inflammatory, mitochondrial, or insulin-signalling biomarkers were not available to directly test mechanistic pathways. Additionally, residual confounding related to lifestyle or cardiometabolic factors cannot be fully excluded. Longitudinal analyses will be essential to determine whether IR-related vascular injury and cognitive differences predict accelerated decline over time.

In summary, in this cognitively normal midlife cohort, decreased insulin sensitivity was associated with greater frontal small vessel disease and lower global cognitive performance, with evidence that the cognitive association is largely direct rather than mediated by vascular burden, hippocampal atrophy, or global perfusion. These findings position decreased insulin sensitivity as a multi-system correlate of early brain vulnerability, associated with partially parallel vascular and neural pathways during midlife. Whether targeting metabolic dysfunction during this window helps preserve long-term cognitive health and reduce later-life neurodegenerative risk warrants testing in longitudinal and interventional studies.

## Contributors

Rohith Erukulla, Graham Reid, and Ivan Koychev conceived and designed the study. Rohith Erukulla drafted the manuscript. Graham Reid, Courtney Yotter, Maria-Elini Dounavi, Audrey Low, John O'Brien, Craig Ritchie, Brian Lawlor, Lorina Naci, Paresh Malhotra, and Ivan Koychev reviewed the manuscript. Brian Lawlor, Lorina Naci, and Paresh Malhotra collected the data. Maria-Elini Dounavi, Audrey Low, and John O'Brien processed the images. Rohith Erukulla and Graham Reid analysed the data. Graham Reid and Ivan Koychev verified the data. All authors agreed to be held accountable for all aspects of this work and approved the final version of the manuscript.

## Data sharing statement

Data related to this study are available within the text, figures and tables. All are available from the corresponding author upon request. The PREVENT dataset is available to access through a data request on the study website (www.preventdementia.co.uk); on the Alzheimer's Disease Data Initiative (ADDI) platform baseline dataset.

DOI: https://doi.org/10.34688/PREVENTMAIN_BASELINE_700V1; Dementia Platforms UK (DPUK); and the Global Alzheimer's Association Network (GAAIN).

## Declaration of interests

P.M. reports research funding from the Alzheimer's Society; grants or contracts from the National Institute for Health and Care Research, FIFA, the Football Association, Alzheimer's Research UK, the Medical Research Council, Dementias Platform UK, LifeArc, and the UK Dementia Research Institute; participation in a Johnson & Johnson Data Safety Monitoring Board; service on the Alzheimer's Society Board of Trustees; a role as the NIHR Research Delivery Network National Specialty Lead for Dementia and Neurodegeneration; and receipt of study drugs through a drugs-only grant from Shire/Takeda Pharmaceuticals. B.L. reports membership of the TOP HAT Data Safety Monitoring Board and leadership or fiduciary roles on the ACCM and MIRA Boards. L.N. reports funding from a Health Research Board Ireland Individual-Led Project Grant, ILP-POR-2024-057. M-E.D. reports an Alzheimer's Society-funded postdoctoral research fellowship and a travel grant from the Guarantors of Brain to attend the 2023 ISMRM scientific meeting in Toronto. C.R. is Chief Executive Officer, founder and majority shareholder of Scottish Brain Sciences, which holds or has held contracts with Lilly, Roche, Roche Diagnostics, Therini, MSD, Merck, Actinogen, Eisai, EXCIVA, Novartis, Janssen Cilag, and Linus Health; no related fees or honoraria were paid to C.R. personally. J.T.O. reports consultancy fees from Biogen, Roche, GE Healthcare and Okwin; lecture honoraria from GE Healthcare; participation on advisory boards for DSMB, TauRx and Novo Nordisk; service as Chair of the UK Alzheimer's Society Research Strategy Council; and academic research support from Avid/Lilly, Merck, UCB, and Alliance Medical. I.K. reports salary support from the UK Dementia Research Institute; investigator-initiated grant funding from Novo Nordisk; grant funding from Alzheimer's Research UK, the Alzheimer's Society, the National Institute for Health and Care Research, the READ-OUT Blood Biomarker Challenge and the Medical Research Council Dementias Platform UK; roles as Head of Clinical Translation at Prima Mente, Chief Medical Officer at Five Lives, and Medical Director at Oxford Brain Diagnostics; consultancy for Johnson & Johnson; speaker fees from Eisai and Novo Nordisk for non-promotional educational events; provision of Expert evidence in neuropsychiatry to UK- and Ireland-based law firms; membership of the NIHR Efficacy and Mechanism Evaluation Committee; and personal stock options in Prima Mente, Five Lives, and Reminiscentia Inc. A.L., C.Y., G.R., and R.E. declare no competing interests.

## References

[1] Savelieff M.G., Chen K.S., Elzinga S.E., Feldman E.L. (2022). Diabetes and dementia: clinical perspective, innovation, knowledge gaps. J Diabetes Complications.

[2] Livingston G., Huntley J., Liu K.Y. (2024). Dementia prevention, intervention, and care: 2024 report of the Lancet standing Commission. Lancet.

[3] Cholerton B., Baker L.D., Craft S. (2013). Insulin, cognition, and dementia. Eur J Pharmacol.

[4] Kullmann S., Heni M., Hallschmid M., Fritsche A., Preissl H., Häring H.-U. (2016). Brain insulin resistance at the crossroads of metabolic and cognitive disorders in humans. Physiol Rev.

[5] Hallschmid M. (2021). Intranasal Insulin for Alzheimer's Disease. CNS Drugs.

[6] Reger M.A., Watson G.S., Green P.S. (2008). Intranasal insulin improves cognition and modulates beta-amyloid in early AD. Neurology.

[7] de la Monte S.M., Wands J.R. (2008). Alzheimer's disease is type 3 diabetes—evidence reviewed. J Diabetes Sci Technol.

[8] Karvani M., Simos P., Stavrakaki S., Kapoukranidou D. (2019). Neurocognitive impairment in type 2 diabetes mellitus. Hormones.

[9] Prins N.D., Scheltens P. (2015). White matter hyperintensities, cognitive impairment and dementia: an update. Nat Rev Neurol.

[10] Tamura Y., Araki A. (2015). Diabetes mellitus and white matter hyperintensity. Geriatr Gerontol Int.

[11] Poels M.M., Ikram M.A., Vernooij M.W. (2008). Total cerebral blood flow in relation to cognitive function: the Rotterdam Scan Study. J Cereb Blood Flow Metab.

[12] Weijs R.W.J., Shkredova D.A., Brekelmans A.C.M., Thijssen D.H.J., Claassen J.A.H.R. (2023). Longitudinal changes in cerebral blood flow and their relation with cognitive decline in patients with dementia: current knowledge and future directions. Alzheimers Dement.

[13] Mirowsky J., Ross C.E. (1992). Age and depression. J Health Soc Behav.

[14] Sharma R., Sekhon S., Cascella M. (2022). http://www.ncbi.nlm.nih.gov/books/NBK562167/.

[15] Ritchie C.W., Ritchie K. (2012). The PREVENT study: a prospective cohort study to identify mid-life biomarkers of late-onset Alzheimer's disease. BMJ Open.

[16] Ritchie C.W., Bridgeman K., Gregory S. (2024). The PREVENT dementia programme: baseline demographic, lifestyle, imaging and cognitive data from a midlife cohort study investigating risk factors for dementia. Brain Commun.

[17] Gayoso-Diz P., Otero-González A., Rodriguez-Alvarez M.X. (2013). Insulin resistance (HOMA-IR) cut-off values and the metabolic syndrome in a general adult population: effect of gender and age: EPIRCE cross-sectional study. BMC Endocr Disord.

[18] Ritchie K., de Roquefeuil G., Craig R. (2014). COGNITO: computerized assessment of information processing. J Psychol Psychother.

[19] Whiteside D.M., Kealey T., Semla M. (2016). Verbal fluency: language or executive function measure?. Appl Neuropsychol Adult.

[20] Chan D., Gallaher L.M., Moodley K., Minati L., Burgess N., Hartley T. (2016). The 4 mountains test: a short test of spatial memory with high sensitivity for the diagnosis of pre-dementia Alzheimer's disease. J Vis Exp.

[21] Dounavi M.-E., Newton C., Jenkins N. (2022). Macrostructural brain alterations at midlife are connected to cardiovascular and not inherited risk of future dementia: the PREVENT-dementia study. J Neurol.

[22] Dounavi M.-E., Mak E., Swann P. (2023). Differential association of cerebral blood flow and anisocytosis in APOE ε4 carriers at midlife. J Cereb Blood Flow Metab.

[23] Low A., Prats-Sedano M.A., McKiernan E. (2022). Modifiable and non-modifiable risk factors of dementia on midlife cerebral small vessel disease in cognitively healthy middle-aged adults: the PREVENT-dementia study. Alzheimers Res Ther.

[24] Chappell M.A., Groves A.R., Whitcher B., Woolrich M.W. (2009). Variational Bayesian inference for a nonlinear forward model. IEEE Trans Signal Process.

[25] Buxton R.B., Frank L.R., Wong E.C., Siewert B., Warach S., Edelman R.R. (1998). A general kinetic model for quantitative perfusion imaging with arterial spin labeling. Magn Reson Med.

[26] Chappell M.A., Groves A.R., MacIntosh B.J., Donahue M.J., Jezzard P., Woolrich M.W. (2011). Partial volume correction of multiple inversion time arterial spin labeling MRI data. Magn Reson Med.

[27] Reuter M., Schmansky N.J., Rosas H.D., Fischl B. (2012). Within-subject template estimation for unbiased longitudinal image analysis. NeuroImage.

[28] Brugulat-Serrat A., Salvadó G., Sudre C.H. (2020). Patterns of white matter hyperintensities associated with cognition in middle-aged cognitively healthy individuals. Brain Imaging Behav.

[29] Boutzoukas E.M., O'Shea A., Albizu A. (2021). Frontal white matter hyperintensities and executive functioning performance in older adults. Front Aging Neurosci.

[30] Yang X., Zhang S., Dong Z. (2019). Insulin resistance is a risk factor for overall cerebral small vessel disease burden in old nondiabetic healthy adult population. Front Aging Neurosci.

[31] Ryu S.Y., Coutu J.-P., Rosas H.D., Salat D.H. (2014). Effects of insulin resistance on white matter microstructure in middle-aged and older adults. Neurology.

[32] Nagayama T., Yamaguchi S., Nakayama T., Yang S., Inagaki S., Nagayama M. (2023). Effects of white matter hyperintensities on isolated executive function assessed by the trail making test. F1000Res.

[33] Toppala S., Ekblad L.L., Lötjönen J. (2019). Midlife insulin resistance as a predictor for late-life cognitive function and cerebrovascular lesions. J Alzheimers Dis.

[34] Atabi F., Moassesfar M., Nakhaie T., Bagherian M., Hosseinpour N., Hashemi M. (2025). A systematic review on type 3 diabetes: bridging the gap between metabolic dysfunction and Alzheimer's disease. Diabetol Metab Syndr.

[35] Meng X., D'Arcy C. (2012). Education and dementia in the context of the cognitive reserve hypothesis: a systematic review with meta-analyses and qualitative analyses. PLoS One.

[36] Farruggia M.C., Small D.M. (2019). Effects of adiposity and metabolic dysfunction on cognition: a review. Physiol Behav.

[37] Stranahan A.M., Norman E.D., Lee K. (2008). Diet-induced insulin resistance impairs hippocampal synaptic plasticity and cognition in middle-aged rats. Hippocampus.

[38] Wang Y., Sun L., He G. (2021). Cerebral perfusion alterations in type 2 diabetes mellitus - a systematic review. Front Neuroendocrinol.

